# Robust GPS/Visual–Inertial Navigation in Dynamic and GPS-Degraded Environments via Dynamic Feature Masking and GPS Measurement Screening

**DOI:** 10.3390/s26217094

**Published:** 2026-11-09

**Authors:** Wei Yi, Hongwei Bian, Changding Xu, Rongying Wang, Shuai Zhao, Xin Chen

**Affiliations:** 1 College of Electrical Engineering, Naval University of Engineering, Wuhan 430033, China; ewen2026@163.com (W.Y.);; 2 Qiushi Academy for Advanced Studies, Zhejiang University, Nanchang 330036, China

**Keywords:** integrated navigation, visual–inertial odometry, dynamic feature masking, GPS measurement screening

## Abstract

**Highlights:**

- A lightweight GPS/VIO framework is developed to improve measurement reliability without modifying the underlying VIO estimator.
- A YOLOv8-based asynchronous dynamic mask reduces the influence of potentially dynamic visual features in traffic scenes.
- GPS LLA measurements are transformed into ENU and aligned with the VIO frame using an initial common trajectory.
- A two-stage GPS screening strategy combines receiver-reported covariance and NIS consistency testing.
- Field experiments demonstrate improved positioning performance in dynamic traffic and reduced trajectory disturbances under GPS-degraded conditions.

**What are the main findings?**

- Field experiments in dynamic traffic and GPS-degraded urban canyons yield three core findings. First, YOLOv8-based dynamic masking reduces horizontal RMSE (2.074 m to 1.820 m) and maximum horizontal error (17.823 m to 11.963 m), effectively suppressing dynamic visual interference. Second, the two-stage GPS screening decreases trajectory jitter from 0.0425 m to 0.0218 m and reduces apparent positional and kinematic disturbances indicated by the selected trajectory and kinematic metrics. Third, the proposed measurement-level processing improves GPS/VIO robustness without modifying the underlying VIO estimator.

**What are the implications of the main findings?**

- This work has both methodological and practical implications. Methodologically, it proposes a simple, generalizable measurement-level optimization paradigm, avoiding cumbersome estimator reconstruction for multi-sensor fusion. Practically, the integrated masking and screening scheme improves the robustness of GPS/VIO navigation under the evaluated dynamic-traffic and GPS-degraded conditions. The measurement-level design provides a practical implementation strategy for improving GPS/VIO robustness without reconstructing the underlying estimator, while its extension to other multi-sensor navigation systems remains to be further validated.

**Abstract:**

Achieving reliable localization in dynamic environments remains challenging because moving objects corrupt visual constraints, and degraded GPS measurements introduce erroneous absolute-position corrections. To address these challenges, this paper presents a robust GPS/visual–inertial navigation (VIO) framework that enhances the reliability of visual and GPS measurements at the measurement level without modifying the underlying VIO estimator. In the visual front end, a YOLOv8-based detector asynchronously processes downsampled images to identify potentially dynamic regions, which are subsequently mapped back to the original resolution to generate a dynamic mask. Features detected within these masked regions are excluded from subsequent visual state estimation. For GPS integration, receiver-provided latitude, longitude, and altitude (LLA) coordinates are first transformed into a local East-North-Up (ENU) frame. Temporally aligned initial segments of the GPS and VIO trajectories are then used to estimate the relative yaw rotation between the two coordinate frames, thereby expressing GPS positions directly within the VIO frame. Prior to each GPS update, a two-stage screening strategy combining receiver-reported covariance with normalized innovation squared (NIS) testing rejects position measurements exhibiting excessive uncertainty or statistical inconsistency with the VIO state prediction. Vehicle field experiments in a dynamic-traffic environment demonstrate that dynamic-feature masking reduces the horizontal RMSE from 2.074 m to 1.820 m and the maximum horizontal error from 17.823 m to 11.963 m. In a GPS-degraded urban-canyon scenario, the proposed screening strategy reduces trajectory jitter from 0.0425 m to 0.0218 m while mitigating positional and kinematic disturbances, as indicated by the trajectory-jitter and kinematic metrics. These results demonstrate that measurement-level screening improves GPS/VIO localization robustness under the evaluated dynamic-traffic and GPS-degraded conditions. The current validation focuses on ground-vehicle operation in urban traffic and GPS-degraded environments; the effectiveness of the asynchronous masking strategy under highly maneuvering platforms and rapidly changing object motion requires further investigation.

## 1. Introduction

Accurate and reliable localization is essential for autonomous vehicles, mobile robots, and unmanned systems operating in complex environments. Visual–inertial odometry (VIO) has been widely adopted because cameras capture rich environmental geometry and inertial measurement units (IMUs) provide high-rate motion dynamics [1]. However, VIO primarily provides relative motion estimation and may accumulate drift during long-term operation. Global Navigation Satellite System (GNSS) receivers, in contrast, provide globally referenced position measurements that suppress accumulated VIO drift [2]. Nevertheless, GNSS suffers from degraded or unavailable positioning performance in complex scenarios such as urban canyons and occluded areas. Multi-sensor fusion mitigates satellite positioning defects by leveraging complementary observations from heterogeneous sensors. Therefore, integrating GNSS with visual–inertial measurements has become a common strategy for achieving bounded-error, continuous localization.

Numerous GNSS/visual–inertial navigation systems have been developed to leverage these complementary characteristics. GVINS tightly integrates raw GNSS, visual, and inertial measurements within a nonlinear optimization framework, yielding globally referenced state estimation while preserving the high-frequency motion tracking capability of VIO [3,4]. IC-GVINS further introduces an INS-centric architecture that utilizes inertial information throughout the visual processing pipeline, enhancing robustness in complex and highly dynamic environments [5]. Other approaches, such as tightly coupled GNSS-aided visual–inertial localization [6], InGVIO [7], and the point-and-line-feature GPLVINS [8] for aerial platforms, explore alternative filtering and optimization formulations to improve estimation consistency and computational efficiency. More recently, square-root filtering frameworks have been proposed to improve numerical stability and efficiency in GNSS/visual–inertial integration [9]. These studies demonstrate that GNSS integration mitigates long-term VIO drift, thereby improving global localization performance.

Despite these advancements, GNSS/visual–inertial navigation remains susceptible to measurement degradation. In complex environments, GNSS signals are frequently corrupted by multipath propagation, non-line-of-sight (NLOS) signal blockage, adverse satellite geometry, and severe interference. Consequently, researchers have dedicated significant effort to maintaining navigation integrity under degraded or unavailable GNSS conditions [10,11]. For instance, Sky-GVINS employs sky-region segmentation to identify satellites whose line-of-sight (LOS) paths are obstructed by surrounding structures, filtering out compromised GNSS observations in urban canyons [12]. These strategies demonstrate that auxiliary environmental information can identify unreliable GNSS measurements before they are incorporated into the navigation solution. However, these methods typically target specific GNSS error sources or rely exclusively on satellite-level raw observations. When only receiver-level position estimates are available, identifying unreliable individual position fixes remains a major challenge because the quality of the receiver solution cannot be inferred from the navigation message alone [13]. Commercial receivers may output position fixes even when actual errors exceed the reported nominal covariance. Directly incorporating such spurious updates can introduce severe position corruption and trigger trajectory divergence.

Another critical limitation stems from the visual front-end measurements. Conventional VIO algorithms typically assume that the observed scene is predominantly static. However, this assumption is frequently violated in environments with pedestrians, vehicles, and other dynamic entities [14]. Visual features on dynamic objects exhibit image motion that violates epipolar constraints and cannot be attributed solely to camera ego-motion. Retaining these features during tracking introduces corrupt geometric constraints into the optimization, severely degrading state estimation accuracy. This vulnerability is pronounced in urban traffic, where a substantial proportion of tracked features originate from dynamic objects.

To mitigate dynamic-scene interference, previous studies have introduced geometric and semantic approaches to detect and reject dynamic visual observations [15]. Geometric methods identify inconsistent tracks using inter-frame constraints, epipolar geometry with RANSAC, or motion models [16]. While these methods require no additional inference network and impose minimal computational overhead, their performance degrades when ego-motion and object motion are kinematically similar [15,16]. Semantic approaches instead locate dynamic regions with instance segmentation or optical flow [17,18], and recent work couples multi-category semantic segmentation with object tracking to handle multi-object scenarios and partial occlusions [19]. For instance, DynaVINS [20] combines IMU motion priors with geometric constraints to reject feature points associated with continuously dynamic and temporarily static objects, whereas D-VINS [21] fuses semantic masks with epipolar constraints for dynamic feature identification.

Although semantic approaches enhance robustness in dynamic environments, embedding a deep-learning detector directly into a high-frequency VIO pipeline imposes heavy computational overhead. In a synchronous architecture, the visual tracking thread must wait for neural-network inference, reducing the throughput and processing rate of the visual front end [22]. Furthermore, state-of-the-art dynamic VIO systems focus primarily on visual tracking refinement, often neglecting the reliability of absolute positioning measurements within a unified navigation framework. Consequently, two critical measurement-level vulnerabilities persist: dynamic visual features contaminate relative motion estimation, and low-quality GPS measurements inject spurious absolute position updates. Addressing only one source of measurement uncertainty fails to guarantee overall system robustness in complex environments.

Practical GPS/VIO integration also faces a fundamental frame-alignment challenge, which tightly coupled systems using raw GNSS observations often bypass or handle implicitly [23]. Commercial GPS receivers output geodetic coordinates (latitude, longitude, altitude; LLA), whereas VIO operates in an arbitrary local Cartesian frame. Even after converting LLA coordinates to a local East-North-Up (ENU) frame, the horizontal yaw of the ENU frame remains unaligned with the VIO reference frame [24]. Consequently, GPS positions cannot be directly fused as absolute observations without rigorous spatial-temporal frame alignment. For loosely coupled architectures using receiver-level position outputs, establishing this spatial orientation is critical, as GPS coordinates must be expressed in the VIO coordinate system [25].

Based on these observations, GPS/VIO navigation reliability is affected by two distinct measurement-level failure modes: dynamic visual observations corrupt relative motion estimation, and degraded GPS fixes introduce erroneous absolute corrections. Existing studies have addressed these problems from different perspectives, including semantic or geometric dynamic-feature rejection, GNSS quality assessment, and tightly or loosely coupled multi-sensor estimation. However, these approaches generally focus on one measurement source or require modifications to the underlying estimation architecture. In contrast, this work adopts an estimator-agnostic, measurement-level strategy that treats both sources of unreliable observations before they enter the state estimator. Potentially dynamic visual features are excluded using asynchronous semantic masking, while receiver-level GPS measurements are screened based on reported uncertainty and innovation consistency. The underlying VIO propagation, visual estimation, and state-update formulation remain unchanged. Therefore, the contribution of this work lies not in introducing a novel object detector or statistical test, but in developing and evaluating a lightweight, measurement-level robustness architecture for GPS/VIO navigation using only camera, IMU, and receiver-level GPS outputs.

To this end, this paper proposes a robust GPS/visual–inertial navigation framework tailored for dynamic and GPS-degraded environments. A YOLOv8-based detector runs asynchronously alongside the visual–inertial tracking pipeline to identify potentially dynamic regions. A dual-resolution strategy performs object detection on downsampled images, while the visual front end processes high-resolution images to retain fine feature details. The detected bounding boxes are mapped to the original image space to generate a dynamic mask, and features within masked regions are pruned before state estimation. For GPS integration, receiver-provided LLA coordinates are transformed into a local ENU frame. Following VIO initialization, temporally correlated GPS and VIO trajectory segments are used to estimate the relative yaw rotation via least squares, establishing a unified coordinate frame. Additionally, a two-stage screening mechanism evaluates receiver-reported covariance and NIS innovation metrics to filter GPS measurements prior to estimator fusion. Measurements failing either criterion are rejected, allowing the estimator to rely safely on the propagated VIO state and valid visual tracks.

Several recent studies have addressed combined dynamic visual environments and degraded GNSS, representing the closest counterparts to this work. Zhai and Yuan [26] proposed a loosely coupled vision-aided GNSS method in which a Dynamic-SLAM front end suppresses dynamic-object effects and a semantic-based adaptive Kalman filter maintains positioning in GNSS-challenged conditions. Adham et al. developed a smart feature-grading mechanism to overcome cautious and excessive dynamic-feature removal [27], and further embedded semantic-aware and multi-level geometric constraints in a global VINS framework with adaptive multilayer VI-GNSS optimization and subsystem-failure detection [28]. Hu et al. [29] presented a visual–inertial-GNSS positioning system that applies deep-learning-based feature extraction and clustering-based outlier detection to remove dynamic and mismatched visual observations. Wang et al. [30] combined semantic masking with stereo epipolar geometric verification and tightly coupled factor-graph GNSS fusion. These systems achieve strong performance but rely on specifically designed or tightly coupled estimators, and several require raw GNSS observations. The present work differs in positioning: rather than proposing a new estimator or dynamic-feature rejection algorithm, it provides an estimator-agnostic, measurement-level architecture. Visual observations and receiver-level GPS fixes are conditioned before entering an otherwise unchanged VIO estimator, requiring only the position and covariance outputs from standard commercial receivers. Table 1 summarizes the component-by-component comparison between the proposed framework and related studies.

The main contributions of this work are summarized as follows:

- An estimator-agnostic measurement-level robustness framework is developed for GPS/VIO navigation in dynamic and GPS-degraded environments. Instead of modifying the underlying VIO estimator, the proposed framework improves the reliability of visual and GPS measurements before they are incorporated into the state estimation process.
- An asynchronous dynamic-feature masking mechanism is implemented for the visual front end. YOLOv8-based detection is performed independently of visual–inertial tracking, while a dual-resolution strategy reduces the computational burden of semantic inference. Detected potentially dynamic regions are mapped to the original image domain and excluded from subsequent visual feature processing.
- A receiver-level GPS processing pipeline is developed, including LLA-to-ENU conversion, horizontal GPS/VIO yaw alignment, and two-stage measurement screening based on receiver-reported covariance and normalized innovation squared (NIS). The approach requires only receiver-provided position and covariance information and does not rely on raw pseudorange or Doppler observations.

The proposed framework is evaluated using vehicle-mounted field data under both dense dynamic traffic and GPS-degraded urban-canyon conditions. The experiments separately assess the effects of dynamic-feature masking and GPS measurement screening, demonstrating improved positioning performance and reduced trajectory disturbances under the evaluated conditions.

## 2. Materials and Methods

In the remainder of this paper, GNSS refers to satellite navigation systems in general, whereas GPS specifically denotes the receiver-level position measurements provided by the commercial receiver used in this work.

The proposed navigation framework integrates GPS and visual–inertial measurements within an error-state filtering framework. The underlying VIO estimator provides high-rate state propagation and visual motion estimation, while GPS provides an absolute position constraint. In this work, the VIO propagation and visual measurement formulations are retained without modification. Instead, additional processing is introduced at the measurement level to improve the reliability of visual and GPS observations before they are incorporated into the estimator.

**Figure 1:**
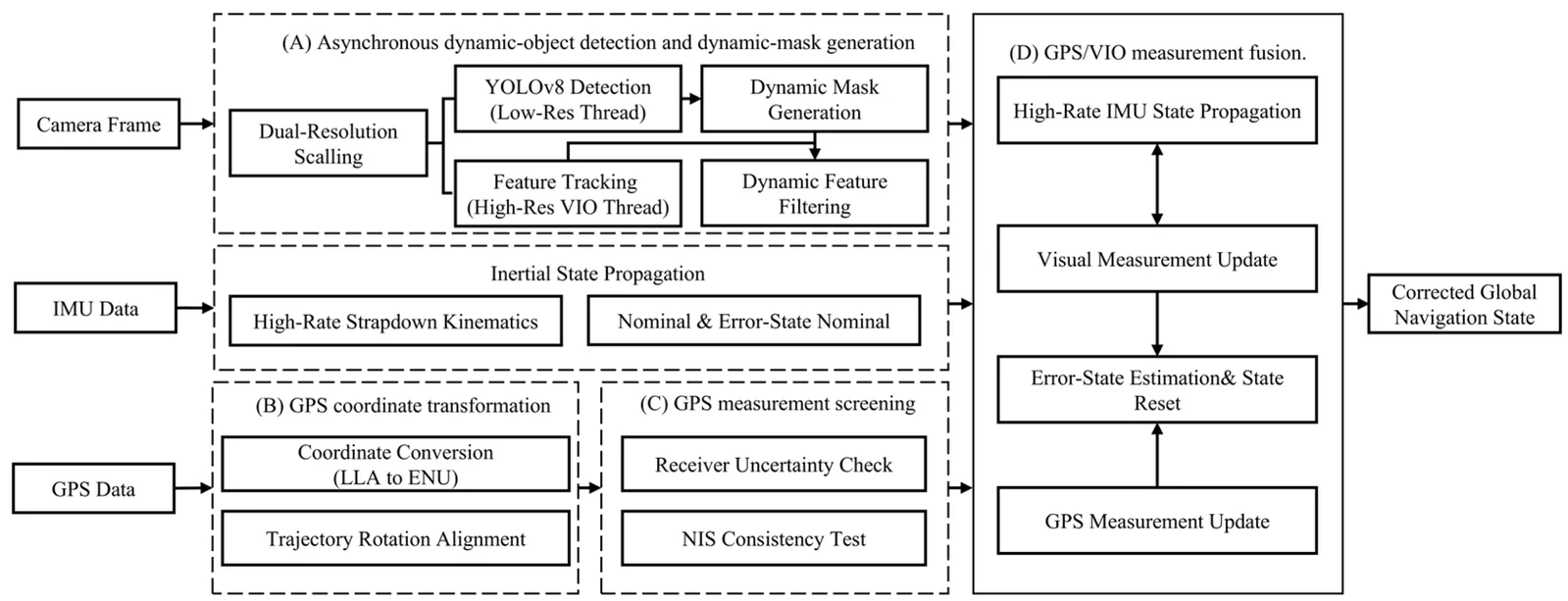
Overall architecture of the proposed GPS/visual–inertial navigation framework with dynamic feature masking and GPS measurement screening.

As shown in Figure 1, the proposed framework consists of four components: (A) asynchronous dynamic-object detection and dynamic-mask generation, (B) GPS coordinate transformation and GPS/VIO frame alignment, (C) GPS measurement screening, and (D) GPS/VIO measurement fusion.

### 2.1. Asynchronous Dynamic-Object Detection and Dynamic Feature Mask Generation

The visual–inertial front-end assumes that tracked visual features primarily originate from static environmental structures. However, features located on moving objects may exhibit motion inconsistent with the camera motion and consequently introduce unreliable visual constraints. To mitigate this problem, a semantic object detection module is introduced to identify potentially dynamic regions before visual features are used for state estimation.

A YOLOv8-based object detector is employed to identify object regions that may contain dynamic visual features. Since deep-learning inference introduces additional computational overhead, object detection is separated from visual–inertial tracking through an asynchronous multi-threaded architecture. The visual–inertial thread continuously processes incoming camera images and performs feature tracking, while an independent detection thread retrieves images and performs YOLOv8 inference. The latest available detection result is asynchronously shared with the visual–inertial thread.

This architecture prevents the visual–inertial tracking process from waiting for neural-network inference and therefore avoids introducing detector latency directly into the high-frequency visual processing loop. Because the camera operates at 20 Hz, the interval between consecutive camera frames is approximately 50 ms, whereas the detector does not necessarily process every camera frame. For each camera frame, the visual–inertial thread uses the most recent available detection result. Let t denote the timestamp of the current camera frame and td the timestamp associated with the latest valid detection result. The age of the dynamic mask is defined as Δt = t − td. In the current implementation, the detector consumes camera frames in a latest-wins manner: each incoming frame overwrites any pending input, and the visual–inertial thread always applies the most recently completed inference result, so that the age of the applied mask is bounded by one detector cycle plus the inference latency, rather than by a fixed software timeout. In the evaluated run, the detector operates at 13.2 Hz with an average inference latency of 20.5 ms. The displacement of a moving object during this interval can be approximated by *v* × Δ*t*, where *v* is the object velocity relative to the camera; the temporal validity of the mask is therefore directly related to the object velocity and the detector latency. This asynchronous design prevents neural-network inference from blocking the visual–inertial thread while keeping the dynamic-region information sufficiently recent for the evaluated vehicle-mounted scenario.

To further reduce the computational cost of semantic inference, the detection branch operates on a downsampled image, while the visual–inertial branch retains the original image resolution. Let the original and detection images have dimensions *W_h_* × *H_h_* and *W_l_* × *H_l_*, respectively. The relationship between the low- and high-resolution image coordinates is

(1) $$u_{h}=\left[\begin{array}{cc} s_{x} & 0\\ 0 & s_{y} \end{array}\right]u_{l}$$

where

(2) $$s_{x}=\frac{W_{h}}{W_{l}},\;s_{y}=\frac{H_{h}}{H_{l}}$$

Here, $u_{l}={\left[u_{l},v_{l}\right]}^{T}$ and $u_{h}={\left[u_{h},v_{h}\right]}^{T}$ denote the pixel coordinates in the low- and high-resolution images, respectively.

The bounding boxes obtained from YOLOv8 are transformed into the original image coordinate system using (1). Let *D_t_* denote the union of detections belonging to predefined potentially dynamic classes at time *t*. The dynamic mask is defined as:

(3) $$M_{t}(u)=\left\{\begin{array}{l} 1,\;u\notin D_{t}\\ 0,\;u\notin D_{t} \end{array}\right.$$

For a tracked visual feature with image coordinate $u_{t}^{i}$, the feature is retained only when

(4) $$M_{t}(u_{t}^{i})=0$$

Accordingly, the valid static feature set is defined as:

(5) $$F_{s}^{s}=\left\{u_{t}^{i}|M_{t}(u_{t}^{i})=0\right\}$$

Features falling inside the detected dynamic regions are therefore removed before they are used to construct visual measurement constraints. The remaining features are processed by the existing visual–inertial estimation framework. This semantic gating mechanism prevents features in detected potentially dynamic regions from entering visual measurement updates while leaving the underlying VIO estimation model unchanged.

### 2.2. GPS Coordinate Transformation and Initial Alignment

The GPS receiver outputs geographic position measurements in latitude, longitude, and altitude (LLA) coordinates. Since VIO estimates position in an independently initialized local Cartesian coordinate frame, GPS measurements must first be transformed into a local coordinate system and subsequently aligned with the VIO frame.

#### 2.2.1. LLA-to-ENU Conversion

Let the first available GPS position be selected as the reference position:

(6) $$l_{0}={\left[\varphi_{0},\lambda_{0},h_{0}\right]}^{T}$$

*φ*_0_, *λ*_0_ and *h*_0_ denote the reference latitude, longitude, and altitude.

For a GPS measurement

(7) $$l_{i}={\left[\varphi_{i},\lambda_{i},h_{i}\right]}^{T}$$

the corresponding ECEF position is first calculated as

(8) $$p_{i}^{\mathrm{ECEF}}=f_{\mathrm{LLA}\to \mathrm{ECEF}}(\varphi_{i},\lambda_{i},h_{i})$$

Taking the ECEF position corresponding to *l*_0_ as the origin, the local ENU coordinate is obtained by

(9) $$p_{i}^{\mathrm{ENU}}=R_{\mathrm{ECEf}}^{\mathrm{ENU}}(p_{i}^{\mathrm{ECEF}}-p_{0}^{\mathrm{ECEF}})$$

where

(10) $$R_{\mathrm{ECEf}}^{\mathrm{ENU}}=\left[\begin{array}{ccc} -\sin\lambda_{0} & \cos\lambda_{0} & 0\\ -\sin\varphi_{0}\cos\lambda_{0} & -\sin\varphi_{0}\sin\lambda_{0} & \cos\varphi_{0}\\ \cos\varphi_{0}\cos\lambda_{0} & \cos\varphi_{0}\sin\lambda_{0} & \sin\varphi_{0} \end{array}\right]$$

Thus, each GPS measurement is represented by

(11) $$p_{i}^{\mathrm{ENU}}={\left[E_{i},N_{i},U_{i}\right]}^{T}$$

#### 2.2.2. Initial GPS-VIO Trajectory Alignment

The ENU frame and the VIO frame have different coordinate orientations. Therefore, the GPS trajectory cannot be directly used as an observation in the VIO coordinate frame.

After VIO initialization, the system simultaneously records the GPS-ENU positions and the corresponding VIO IMU positions. To avoid using measurements during the initial stationary period, the initialization process is started only when the VIO velocity satisfies the prescribed motion condition.

Let the collected GPS and VIO position sequences be $P_{\mathrm{GPS}}=\{p_{1}^{\mathrm{ENU}},\ldots ,p_{N}^{\mathrm{ENU}}\}$ and $P_{\mathrm{VIO}}=\{p_{1}^{\mathrm{VIO}},\ldots ,p_{N}^{\mathrm{VIO}}\}$. The two sequences are paired by timestamp after compensating the VIO timestamps using the estimated camera-to-IMU temporal offset.

To ensure that the trajectory contains sufficient directional information, GPS-VIO alignment is performed only when both the number of collected measurements and the accumulated horizontal displacement satisfy the prescribed thresholds:

(12) $$N\geq N_{\min}$$

and

(13) $$\begin{array}{l} \left\|p_{N}^{\mathrm{ENU},\mathrm{xy}}-p_{1}^{\mathrm{ENU},\mathrm{xy}}\right\|\geq d_{\min}\\ \left\|p_{N}^{\mathrm{VIO},\mathrm{xy}}-p_{1}^{\mathrm{VIO},\mathrm{xy}}\right\|\geq d_{\min} \end{array}$$

where the superscript *xy* denotes the horizontal components, *N*_min_ is the minimum number of initialization measurements, and *d*_min_ is the minimum horizontal displacement.

If the displacement condition is not satisfied, the oldest measurement is removed and the initialization window is continuously advanced. This sliding-window mechanism ensures that the trajectory used for alignment contains sufficient translational motion.

#### 2.2.3. Horizontal Rotation Estimation

The GPS and VIO coordinate frames are aligned by estimating their relative horizontal orientation. Assuming that the ENU up axis and the VIO vertical axis are aligned, the transformation is modeled as a rotation around the vertical axis:

(14) $$p^{\mathrm{VIO}}=R_{\mathrm{ENU}}^{\mathrm{VIO}}p_{\mathrm{ENU}}^{\mathrm{ENU}}$$

where

(15) $$R_{\mathrm{ENU}}^{\mathrm{VIO}}=\left[\begin{array}{ccc} \cos\theta & -\sin\theta & 0\\ \sin\theta & \cos\theta & 0\\ 0 & 0 & 1 \end{array}\right]$$

An initial estimate of *θ* is obtained from the displacement directions of the GPS and VIO trajectories. Define

(16) $$\begin{array}{l} d_{G}=p_{N}^{\mathrm{ENU},\mathrm{xy}}-p_{1}^{\mathrm{ENU},\mathrm{xy}}\\ d_{V}=p_{N}^{\mathrm{VIO},\mathrm{xy}}-p_{1}^{\mathrm{VIO},\mathrm{xy}} \end{array}$$

An initial yaw estimate is computed as:

(17) $$\theta_{0}=\mathrm{atan}2(d_{\mathrm{Gx}}d_{\mathrm{Vy}}-d_{\mathrm{Gy}}d_{\mathrm{Vx}},d_{\mathrm{Gx}}d_{\mathrm{Vx}}+d_{\mathrm{Gy}}d_{\mathrm{Vy}})$$

The rotation angle is subsequently optimized using all simultaneously collected GPS-VIO trajectory pairs. Specifically, the optimal angle is obtained by minimizing the horizontal trajectory alignment error:

(18) $$\theta^{*}=\arg\min_{\theta }\sum_{i=1}^{N}{\left\|p_{i}^{\mathrm{VIO},\mathrm{xy}}-R(\theta )p_{i}^{\mathrm{ENU},\mathrm{xy}}\right\|}^{2}$$

where

(19) $$R(\theta )=\left[\begin{array}{cc} \cos\theta & -\sin\theta \\ \sin\theta & \cos\theta \end{array}\right]$$

The optimized angle is used to construct the final transformation matrix in (15). Consequently, subsequent GPS measurements can be transformed directly into the VIO coordinate frame:

(20) $$p_{\mathrm{GPS}}^{\mathrm{VIO}}=R_{\mathrm{ENU}}^{\mathrm{VIO}}p_{\mathrm{GPS}}^{\mathrm{ENU}}$$

Because both GPS and VIO positions are expressed relative to their respective local origins, the alignment only estimates the relative horizontal rotation and does not introduce an additional translation parameter.

### 2.3. GPS Measurement Screening

Although GPS provides an absolute position constraint, individual GPS position measurements may contain substantial errors. Such errors can arise from multipath propagation, signal attenuation, unfavorable satellite geometry, and other degradation mechanisms. If an erroneous GPS measurement is directly incorporated into the state update, it may introduce a significant position disturbance into the estimated trajectory. Therefore, a measurement-level screening strategy is applied before each GPS update.

The proposed screening strategy considers both the uncertainty reported by the GPS receiver and the statistical consistency between the GPS measurement and the VIO state prediction.

#### 2.3.1. Receiver-Reported GPS Uncertainty

Let the covariance matrix provided by the GPS receiver at time *k* be **R***_GPS_*_,*k*_.

This covariance provides an instantaneous characterization of the uncertainty associated with the GPS position estimate. A measurement with excessively large reported uncertainty is regarded as unreliable and is rejected before the filtering update.

To obtain a scalar measure of the reported horizontal position uncertainty, the horizontal 2 × 2 block of **R***_GPS_*_,*k*_, formed by the east and north components and denoted **R***_h_*_,*k*_, is extracted and its trace is used. Only the horizontal block is evaluated, because the screening targets horizontal position quality, which is the quantity of interest for ground-vehicle navigation; the vertical component is retained in the measurement update but is not used for the covariance gate. Specifically, the overall uncertainty is evaluated using:

(21) $$q_{k}=\mathrm{tr}\left(R_{h,k}\right)$$

The full 3 × 3 matrix **R***_GPS_*_,*k*_ is still used in the position residual, the innovation covariance and the NIS test of Section 2.3.3, where three degrees of freedom are assumed; only the covariance gate operates on the horizontal block.

If *q_k_* > *q*_max_, the GPS measurement is rejected.

This preliminary check excludes measurements for which the receiver reports relatively large position uncertainty before they enter the state estimator.

#### 2.3.2. GPS-VIO Innovation Consistency

Receiver-reported covariance alone may not identify all inconsistent GPS measurements. Therefore, the consistency between the GPS measurement and the current VIO state prediction is further evaluated.

The GPS position residual in the VIO frame is

(22) $$r_{\mathrm{GPS},k}=P_{\mathrm{GPS},k}^{\mathrm{VIO}}-{\hat{p}}_{k}$$

where ${\hat{p}}_{k}$ denotes the predicted IMU position from the VIO state.

The GPS position measurement model is expressed as

(23) $$z_{\mathrm{GPS},k}=p_{k}+n_{\mathrm{GPS},k}$$

where **n***_GPS_*_,*k*_ represents GPS measurement noise.

For the error-state formulation, the corresponding measurement Jacobian is

(24) $$H_{\mathrm{GPS}}=[I_{3}\;0\;0\;0\;0]$$

The innovation covariance is therefore

(25) $$S_{k}=H_{\mathrm{GPS}}P_{k}^{-1}H_{\mathrm{GPS}}^{T}+R_{\mathrm{GPS},k}$$

where $P_{k}^{-1}$ is the predicted error-state covariance.

The normalized innovation squared (NIS) is defined as

(26) $$\gamma_{k}=r_{\mathrm{GPS},k}^{T}S_{k}^{-1}r_{\mathrm{GPS},k}$$

Under the Gaussian measurement-noise assumption, the NIS is approximately chi-square distributed with three degrees of freedom:

(27) $$\gamma_{k}\sim \chi^{2}(3)$$

For a selected false-alarm probability *α*, the measurement is accepted when

(28) $$\gamma_{k}\leq \chi_{3,\alpha }^{2}$$

Conversely, $\gamma_{k}>\chi_{3,\alpha }^{2}$ indicates that the GPS measurement is statistically inconsistent with the current VIO state prediction and is therefore rejected.

#### 2.3.3. Combined GPS Screening Criterion

The final GPS acceptance criterion combines the receiver-reported uncertainty and the GPS-VIO innovation consistency:

(29) $$q_{k}\leq q_{\max}\;\mathrm{and}\;\gamma_{k}\leq \chi_{3,\alpha }^{2}$$

Only GPS measurements satisfying both conditions are used for state correction. Otherwise, the current GPS measurement is discarded, and the estimator continues with the VIO prediction and valid visual measurements.

The two-stage screening mechanism provides complementary criteria for identifying unreliable GPS position measurements. The covariance-based criterion identifies measurements with intrinsically large reported uncertainty, while the NIS criterion detects measurements that are inconsistent with the current navigation state even when the receiver-reported covariance is relatively small.

### 2.4. GPS/VIO Measurement Fusion

After coordinate transformation and measurement screening, accepted GPS measurements are incorporated into the VIO estimator as absolute position constraints. The standard VIO propagation and visual measurement processes are retained without modification; the proposed method operates at the measurement level by screening unreliable visual and GPS observations before they are incorporated into the estimator.

The accepted GPS measurement is expressed in the VIO coordinate frame as

(30) $$z_{\mathrm{GPS},k}=P_{\mathrm{GPS},k}^{\mathrm{VIO}}$$

The corresponding predicted measurement is the position component of the current navigation state:

(31) $${\hat{z}}_{\mathrm{GPS},k}={\hat{p}}_{k}$$

Therefore, the GPS measurement residual is

(32) $$r_{\mathrm{GPS},k}=z_{\mathrm{GPS},k}-{\hat{z}}_{\mathrm{GPS},k}$$

The linearized measurement model is

(33) $$r_{\mathrm{GPS},k}=H_{\mathrm{GPS}}\delta x_{k}+n_{\mathrm{GPS},k}$$

where **H***_GPS_* is given by (24).

For an accepted GPS measurement, the Kalman gain is calculated as

(34) $$K_{k}=P_{k}^{-1}H_{\mathrm{GPS}}^{T}S_{k}^{-1}$$

where

(35) $$S_{k}=H_{\mathrm{GPS}}P_{k}^{-1}H_{\mathrm{GPS}}^{T}+R_{\mathrm{GPS},k}$$

The error-state correction is then obtained as

(36) $$\delta {\hat{x}}_{k}=K_{k}r_{\mathrm{GPS},k}$$

The nominal state is subsequently corrected using the estimated error state, and the covariance is updated according to:

(37) $$P_{k}^{+}=(I-K_{k}H_{\mathrm{GPS}})P_{k}^{-}$$

For measurements rejected by the screening criterion in (29), no GPS measurement update is performed.

Consequently, the proposed framework provides a measurement-level approach for improving the robustness of GPS/VIO fusion. On the visual side, potentially dynamic features are removed before they enter the visual estimator. On the GPS side, measurements with excessive reported uncertainty or statistical inconsistency with the VIO prediction are rejected before state correction. The underlying VIO estimator remains unchanged, which allows the proposed measurement-level modules to be incorporated into an existing visual–inertial navigation framework.

### 2.5. Implementation Details

The implementation parameters used in the experiments are summarized in Table 2 to facilitate reproducibility. For the dynamic-feature masking module, the table reports the YOLOv8 model variant, detection confidence threshold, detector input resolution, original camera resolution, target dynamic object categories, detector execution frequency, and the mask-age statistic. For GPS/VIO alignment, the minimum number of initialization measurements *N*_min_ and the minimum horizontal displacement *d*_min_ are specified together with the motion condition used to activate the alignment procedure. For GPS measurement screening, the covariance threshold *q*_max_ and the false-alarm probability α used for the NIS test are reported. The principal visual–inertial parameters, including the number of tracked features and feature-tracking thresholds, are also provided. The covariance threshold *q*_max_ was selected according to the receiver’s nominal accuracy specifications and is applied to the trace of the horizontal block of the receiver-reported covariance, so that only fixes with clearly abnormal horizontal uncertainty are rejected, and α follows the standard 95% chi-square consistency gate with three degrees of freedom.

## 3. Results and Discussion

### 3.1. Evaluation of Dynamic Feature Masking

#### 3.1.1. Experimental Setup and Test Environment

**Figure 2:**
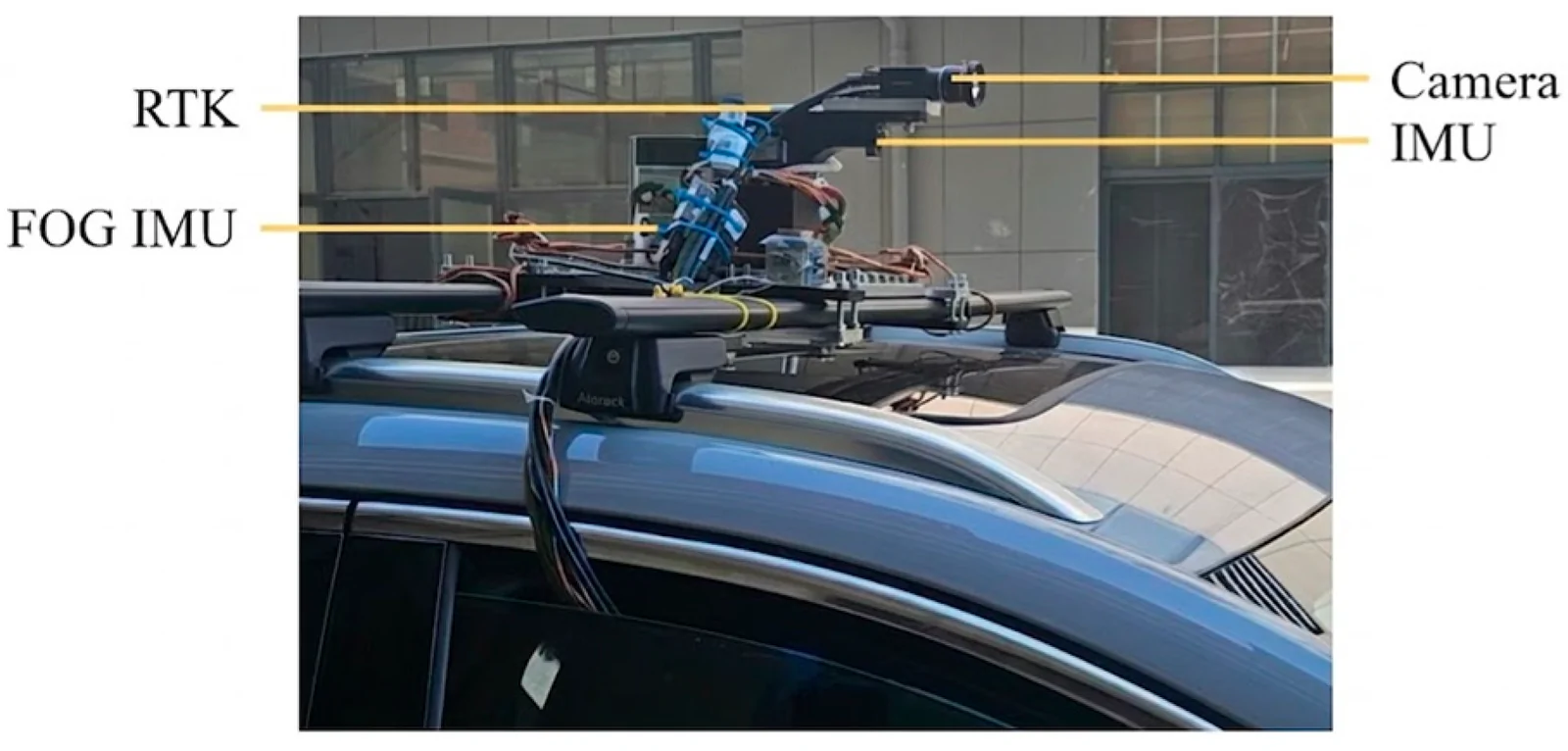
Experimental vehicle and sensor configuration.

The experimental vehicle and sensor configuration are shown in Figure 2. The reference system consists of a high-precision dual-antenna RTK GNSS receiver integrated with a fiber-optic-gyroscope IMU, providing centimeter-level positioning accuracy and sub-degree attitude accuracy and serving as the reference trajectory for performance evaluation. The system under evaluation adopts low-cost consumer-grade sensors, including a monocular global shutter camera capturing images at 20 Hz and a MEMS IMU delivering inertial measurements at 100 Hz.

During the dynamic-traffic experiment, the ego-vehicle traveled at typical urban speeds, while surrounding vehicles moved at comparable speeds and pedestrians at approximately 1–2 m/s. With a 50 ms inter-frame interval, an object with a relative speed of 10 m/s moves approximately 0.5 m between two consecutive frames. The object displacement within the detector inference latency is therefore expected to remain small relative to the detected bounding boxes in the evaluated scenes, so the asynchronously updated mask remains spatially valid for the evaluated dynamics. The GPS and VIO sequences are paired by timestamp with a tolerance of half the GPS output period; the receiver output rate is 5 Hz, measured from the dataset, which gives a tolerance of 100 ms. Pairing is performed after compensating the VIO timestamps using the estimated camera-to-IMU temporal offset.

**Figure 3:**
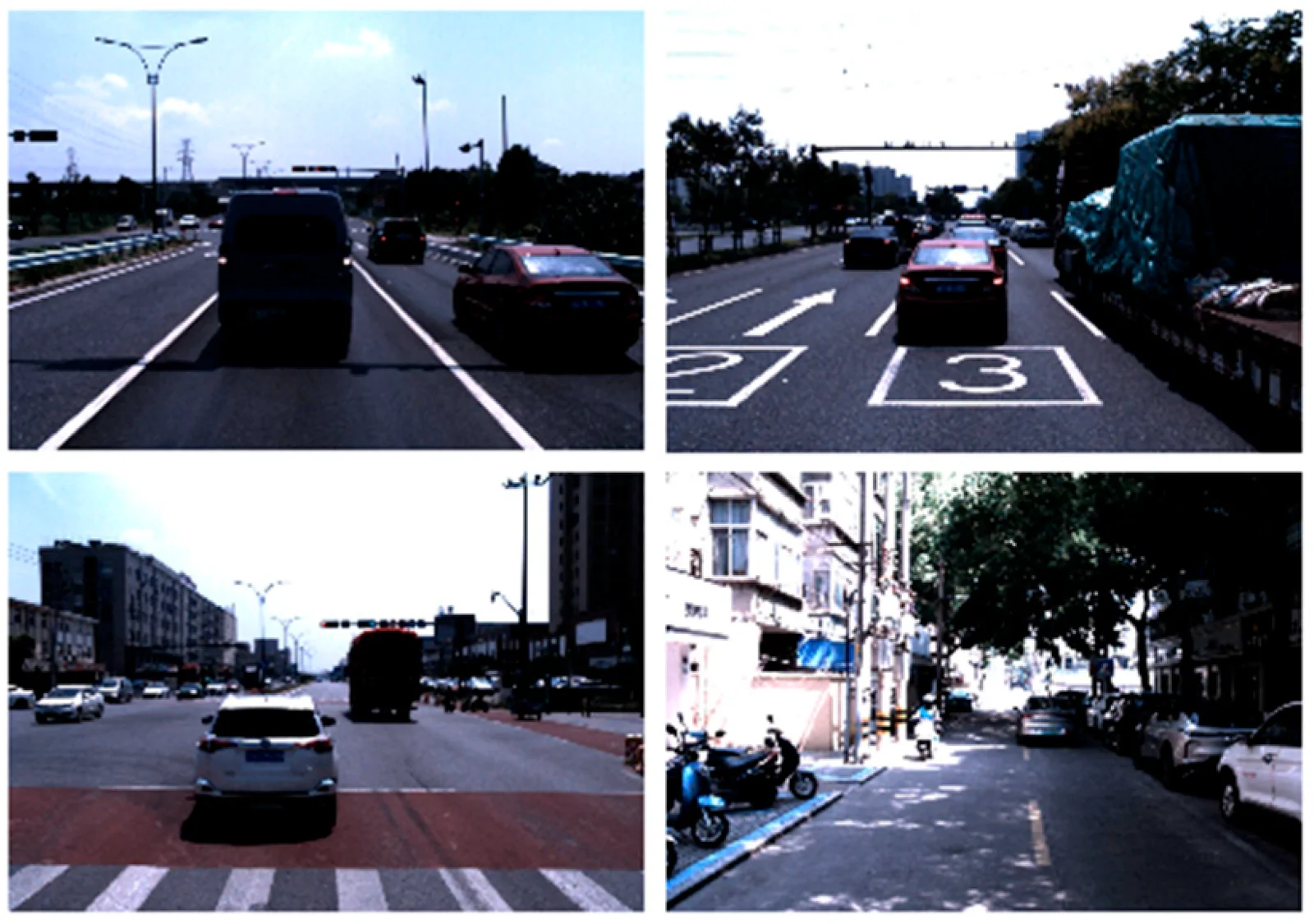
Urban test scenario with dense dynamic traffic.

A representative test route in an urban environment is shown in Figure 3, which contains dense dynamic traffic and pedestrians.

#### 3.1.2. Evaluation Metrics and Pseudo-GPS Construction

To evaluate the navigation system under controlled non-ideal GPS conditions, pseudo-GPS position measurements were constructed from the RTK reference trajectory. Zero-mean Gaussian noise with a standard deviation of 1.5 m was added to the reference positions, together with a constant 5 m bias, to introduce both stochastic and systematic positioning errors. The diagonal elements of the GPS measurement covariance were set to 10, 10, and 20 m^2^ for the east, north, and up components, respectively. The resulting horizontal covariance trace of 20 m^2^ remains below the threshold *q*_max_ given in Table 2, so the covariance gate stays selective rather than rejecting every measurement. The stochastic component represents random receiver-level positioning uncertainty, whose magnitude is consistent with the meter-level horizontal errors of single-point positioning in urban environments, whereas the constant bias provides a controlled systematic error component emulating sustained multipath-induced offsets. This pseudo-GPS model is intended as a controlled stress test rather than a complete statistical model of urban GNSS degradation. In particular, it does not reproduce time-correlated multipath errors, non-line-of-sight effects, satellite-geometry changes, intermittent signal blockage, or abrupt loss of positioning availability. Therefore, the results in this experiment are interpreted as a robustness evaluation under controlled GPS degradation rather than as a comprehensive characterization of real urban-canyon GNSS error statistics. A complementary evaluation with real receiver measurements under naturally occurring signal obstruction is provided in Section 3.2.

#### 3.1.3. Evaluation of VIO and GPS/VIO Performance

Four experimental configurations were evaluated: pure VIO, VIO with dynamic feature masking, GPS-integrated VIO, and GPS-integrated VIO with dynamic feature masking.

**Figure 4:**
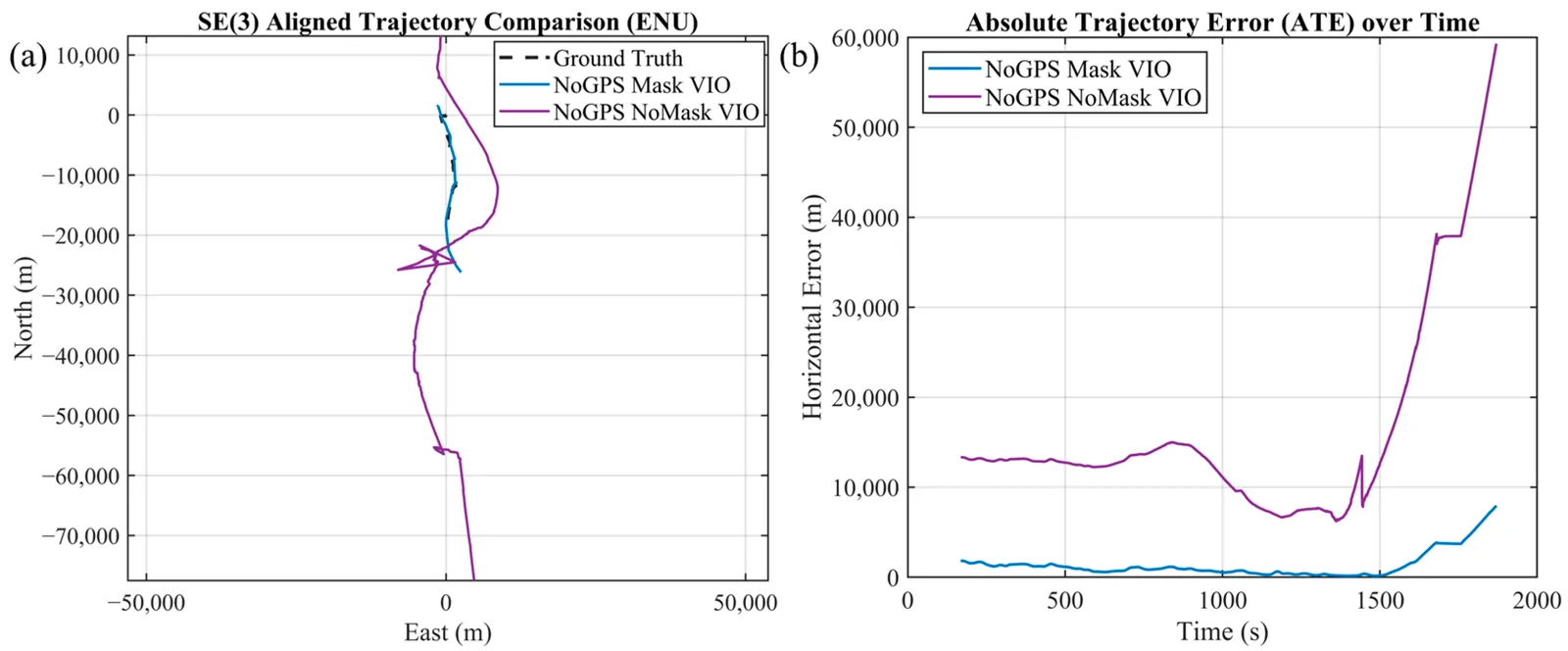
VIO trajectory and absolute-position error comparison with and without dynamic feature masking: (**a**) aligned 2-D trajectories and (**b**) time-series absolute-position errors.

The impact of dynamic feature masking was first evaluated without GNSS aiding. Figure 4a presents the aligned trajectories, while Figure 4b shows the absolute trajectory error over time. In the baseline configuration without dynamic masking, designated as NoGPS NoMask VIO, features associated with potentially dynamic objects, such as vehicles and pedestrians, were retained as visual observations during feature tracking and subsequent state estimation. The resulting trajectory exhibited severe drift, with a horizontal RMSE of 18,867.152 m and a maximum horizontal error of 59,294.336 m. This large error should not be interpreted as being caused exclusively by dynamic objects, since standalone VIO degradation may also result from the accumulation of estimation errors, weak texture, and the limited availability of reliable static features. Nevertheless, enabling dynamic feature masking substantially reduced the observed drift. In the NoGPS Mask VIO configuration, the horizontal RMSE decreased to 1909.592 m and the maximum horizontal error decreased to 7943.267 m. These results indicate that removing potentially dynamic visual observations substantially improves the stability of the evaluated VIO trajectory, although considerable residual drift remains without absolute-position aiding. This residual drift is the error source that the GPS integration described in Section 2 is designed to bound. This unaided case is included to illustrate the sensitivity of the visual front end, not as the intended operating mode of the integrated system.

#### 3.1.4. Integrated GPS/VIO Performance Evaluation

**Figure 5:**
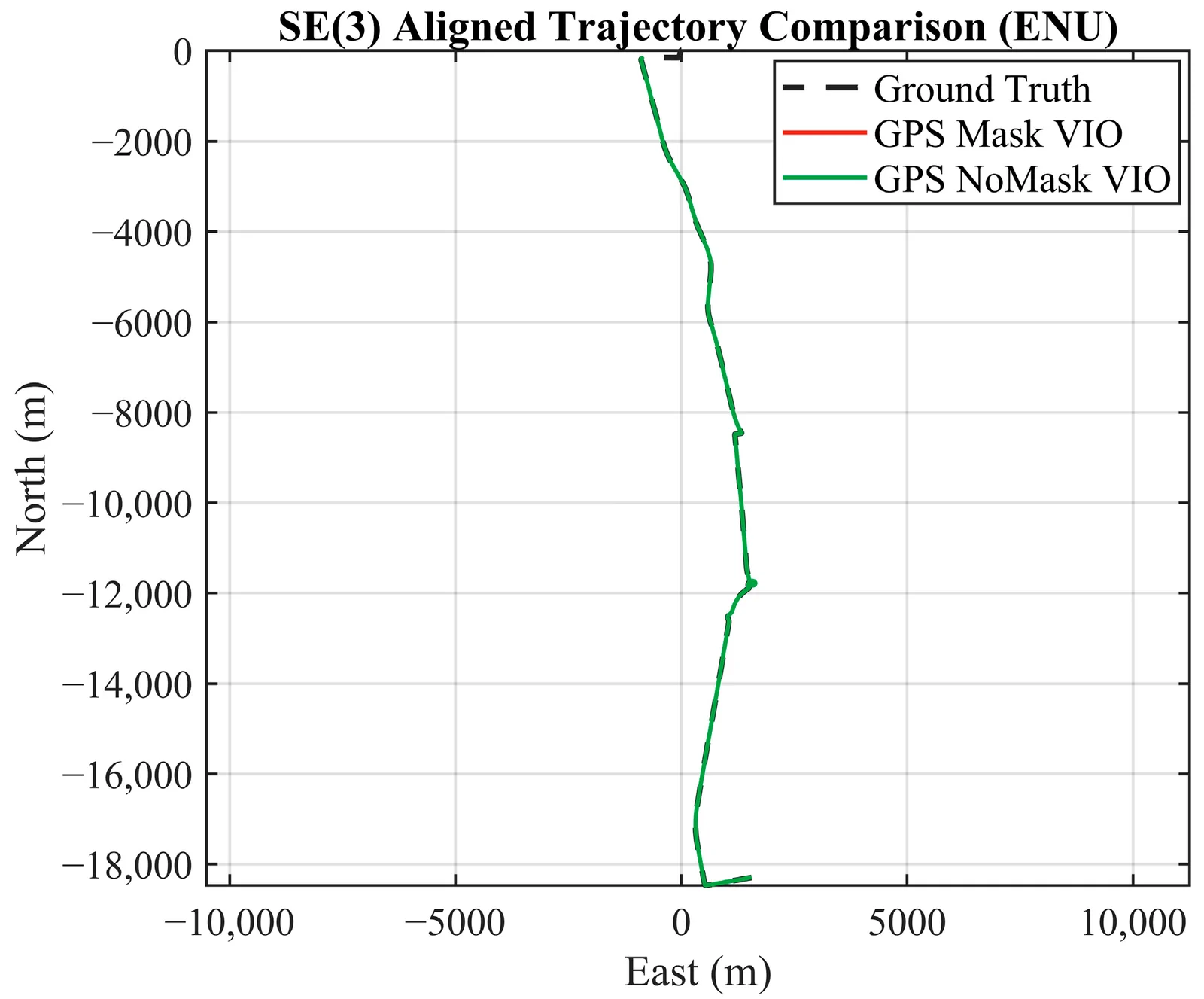
Comparison of GPS/VIO estimated trajectories with and without dynamic feature masking against the reference trajectory.

The performance of the integrated system with GPS aiding was further analyzed. As shown in Figure 5, both GPS/VIO configurations follow the reference trajectory, while the configuration with dynamic feature masking exhibits fewer local trajectory deviations.

The quantitative comparison in Table 3 shows a clear difference in positioning accuracy between the two configurations. Without dynamic masking, the GPS NoMask VIO configuration exhibited local pose fluctuations associated with potentially dynamic visual observations, resulting in a horizontal RMSE of 2.074 m, a 3-D RMSE of 2.133 m, and a maximum horizontal error of 17.823 m. By incorporating dynamic feature masking in the GPS Mask VIO configuration, the visual constraints became less affected by potentially dynamic observations. The integrated system achieved a horizontal RMSE of 1.820 m, a 12.2% reduction, with a 3-D RMSE of 1.897 m, and a reduced maximum horizontal error of 11.963 m, representing a 32.9% reduction. These results indicate that dynamic feature masking reduces the influence of potentially dynamic visual observations and provides an additional accuracy improvement for the GPS/VIO system in dynamic environments.

**Figure 6:**
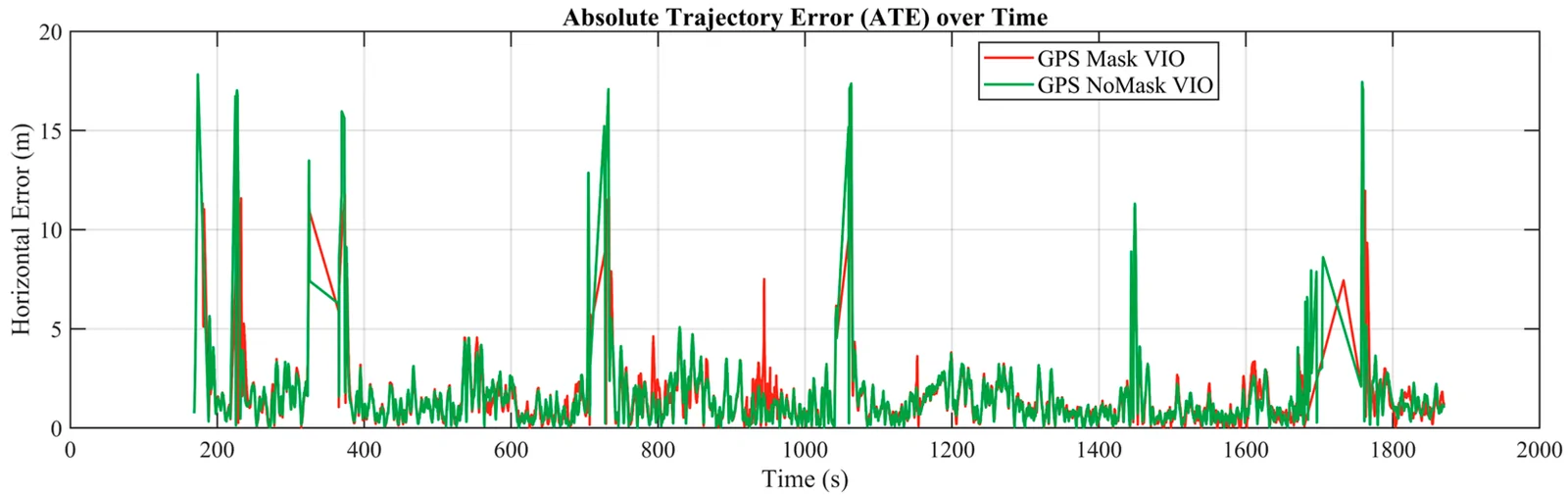
Transient error spike analysis during vehicle stop-and-go intervals at traffic lights.

Furthermore, as shown in Figure 6, intermittent spikes are observed in the estimation error profile. Correlating these spikes with time-series data indicates that the error peaks are mainly observed around stop-and-go intervals at traffic lights. During these intervals, a relatively high density of vehicles was present within the camera field of view. At the moment of re-acceleration, the visual front-end may have been affected by the increased presence of potentially dynamic visual observations, which coincides with the observed transient error amplification. As subsequent GPS updates became available, the accumulated position deviation was progressively reduced, and the estimated trajectory subsequently recovered.

### 3.2. Evaluation of GPS Measurement Screening

#### 3.2.1. Experimental Setup and Urban Canyon Environment

**Figure 7:**
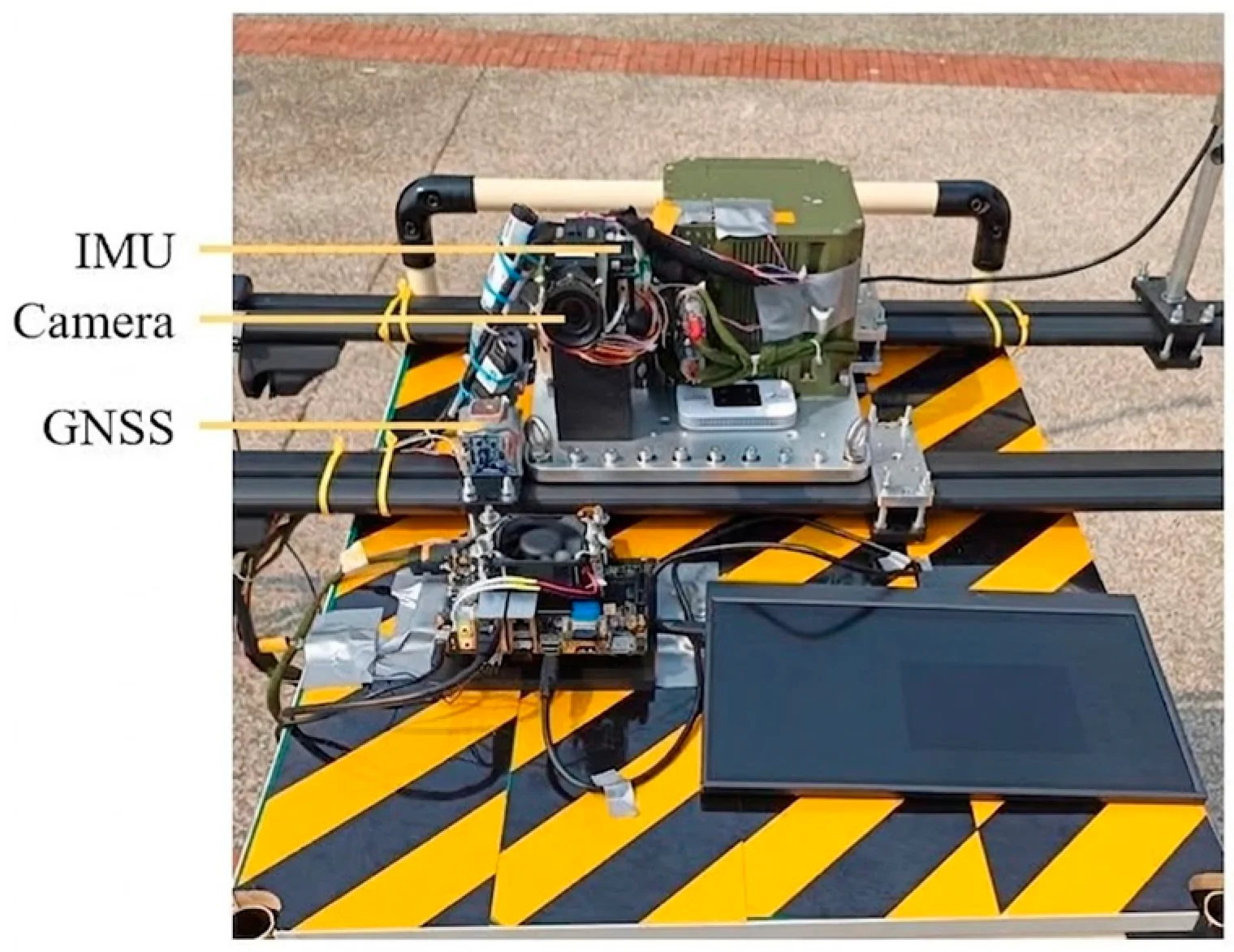
Experimental vehicle and sensor configuration for the GPS-degraded environment test.

Following the validation of visual front-end dynamic feature masking in Section 3.1, this experiment evaluates the performance of the proposed GPS measurement screening strategy under severe signal degradation. Testing was conducted using the sensor configuration shown in Figure 7 along a narrow street bordered by tall structures.

**Figure 8:**
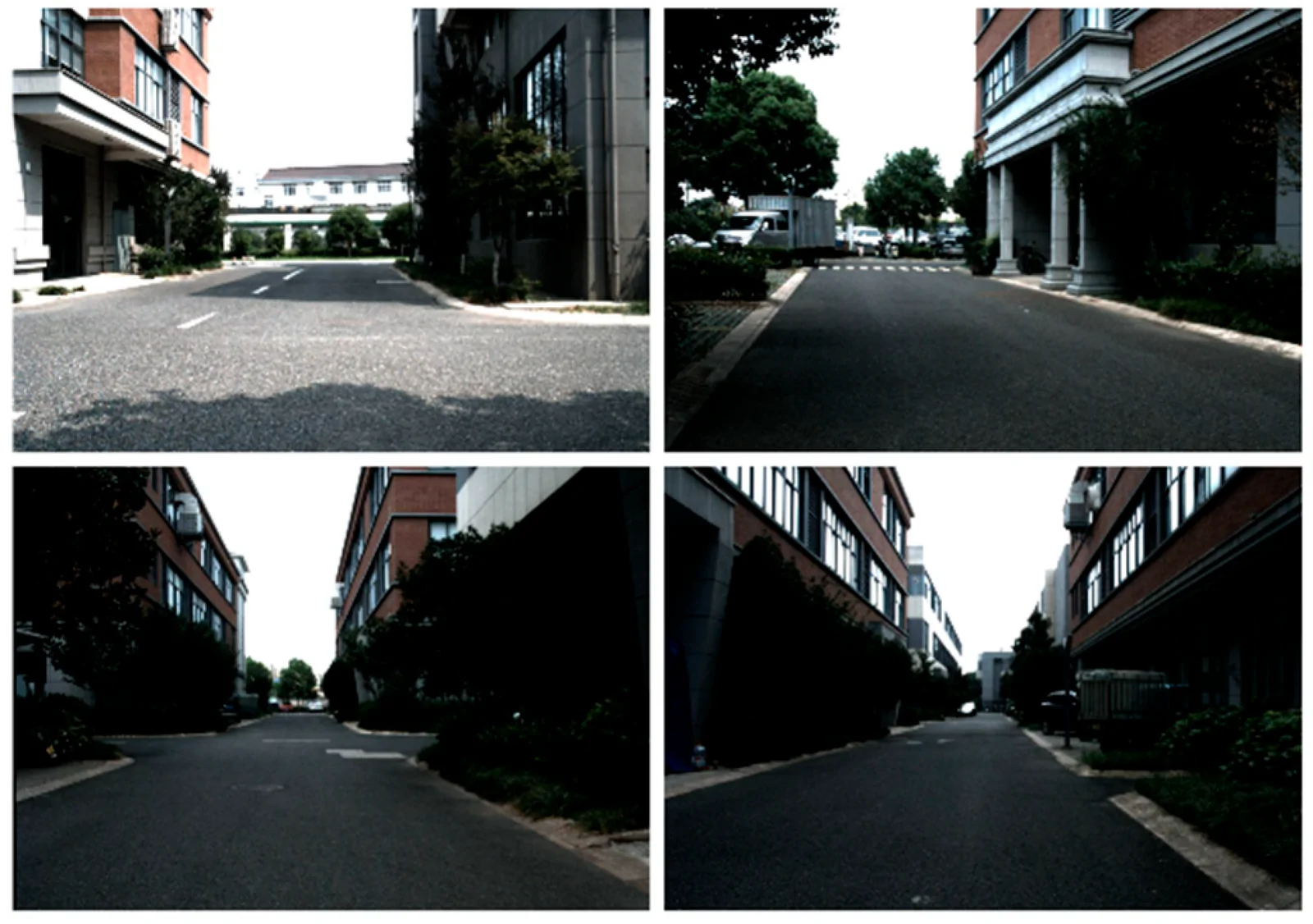
Narrow-street urban canyon environment with severe GPS signal obstruction.

This setup represents a challenging urban canyon environment where severe multipath effects, signal attenuation, and partial line-of-sight occlusions, as illustrated in Figure 8, can introduce large errors into the receiver-provided GPS position measurements. Given the relatively low dynamic traffic density in this scenario, dynamic feature masking was deactivated to isolate the effect of GPS measurement screening. The direct-fusion configuration serves as the baseline and directly incorporates the receiver-provided GPS position measurements into the visual–inertial estimator without additional measurement screening. Both configurations use the same receiver-provided GPS position measurements; the difference is that the proposed configuration performs covariance- and NIS-based screening before the GPS update, whereas the direct-fusion baseline incorporates the measurements without screening. The absolute accuracy of the integrated architecture was benchmarked against RTK reference data in Section 3.1. The same reference system cannot play that role in this section: the test street is flanked by tall buildings, the number of satellites available to the reference receiver drops sharply, and its own position solution degrades under the very conditions being evaluated, so it is no longer of centimeter-level accuracy. No independent reference of comparable quality is therefore available for this scenario, and the evaluation focuses on trajectory smoothness and kinematic disturbances.

#### 3.2.2. Evaluation Metrics and Kinematic Consistency

In the absence of absolute reference trajectories, performance assessment focuses on characterizing trajectory smoothness and kinematic disturbances in the estimated motion state. To this end, total path length, maximum inferred speed, maximum and mean acceleration magnitudes, and trajectory jitter are selected as the primary quantitative metrics. Total path length captures path inflation resulting from high-frequency positional deviations. Maximum speed and acceleration are used to characterize abrupt kinematic disturbances in the estimated trajectory. Trajectory jitter quantifies local trajectory smoothness by calculating the standard deviation of spatial residuals relative to a moving-average baseline.

#### 3.2.3. Fusion Results and Outlier Rejection Analysis

**Figure 9:**
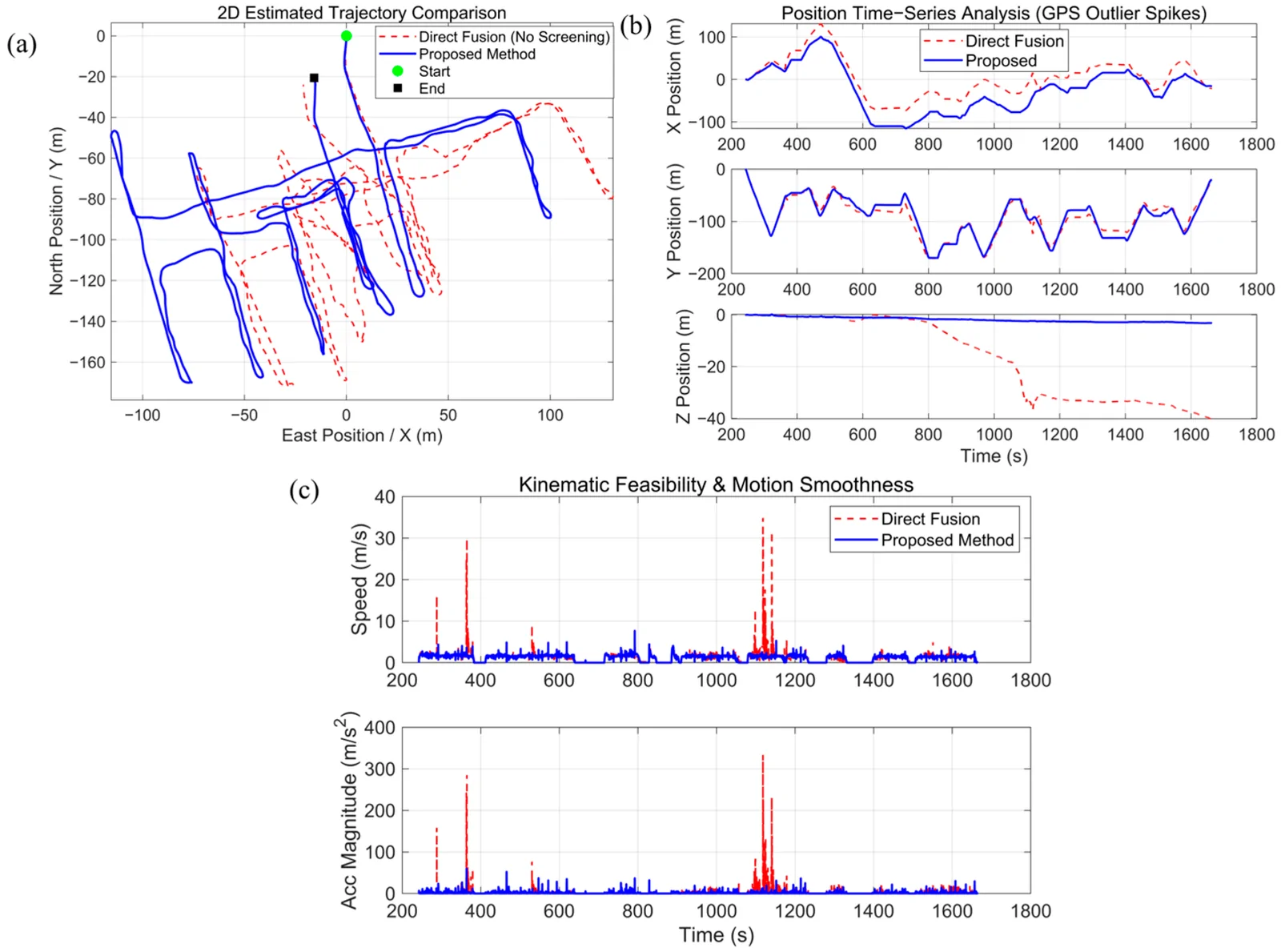
Trajectory and kinematic comparison between direct GPS/VIO fusion and the proposed GPS measurement screening method under GPS-degraded conditions: (**a**) 2D trajectory comparison; (**b**) three-axis position profiles; and (**c**) velocity and acceleration profiles.

Figure 9 and Table 4 summarize the estimated trajectory profiles and quantitative kinematic metrics for the direct-fusion baseline and the proposed method under GPS-degraded conditions. As shown in Figure 9a, direct fusion exhibits pronounced trajectory disturbances when the vehicle traverses the building-obstructed region, consistent with degraded GPS position measurements. The *X*-, *Y*-, and *Z*-axis position profiles in Figure 9b further reveal frequent coordinate spikes in the direct-fusion estimates during GPS-degraded intervals. In terms of kinematic profiles, Figure 9c shows that direct fusion produces a maximum inferred speed of 34.82 m/s and a peak acceleration of 333.52 m/s^2^, indicating pronounced disturbances in the estimated trajectory. Furthermore, the degraded GPS position measurements are associated with increased local trajectory jaggedness, resulting in a total path length of 1784.16 m, a mean acceleration of 2.26 m/s^2^, and a jitter metric of 0.0425 m.

In contrast, the proposed GPS measurement screening strategy screens degraded GPS measurements using receiver-reported uncertainty and GPS/VIO innovation consistency before the state update. As demonstrated in Figure 9a, the trajectory generated by the proposed method exhibits substantially fewer abrupt deviations, with the pronounced position spikes observed in the direct-fusion solution substantially reduced in Figure 9b. Under identical motion sequences, Figure 9c confirms that the proposed method reduces the maximum inferred speed to 7.65 m/s and reduces the peak acceleration to 59.63 m/s^2^. The mean acceleration decreases from 2.26 m/s^2^ to 1.10 m/s^2^, indicating substantially reduced kinematic disturbances compared with direct fusion. Additionally, by reducing the influence of abrupt GPS-induced position deviations, the proposed method reduces the total estimated path length from 1784.16 m to 1697.52 m and suppresses trajectory jitter to 0.0218 m, corresponding to a 48.7% reduction relative to direct fusion. These quantitative findings indicate that the proposed screening module reduces the influence of unreliable GPS measurements on the state estimator, resulting in smoother trajectories and smaller kinematic disturbances.

The validity of using trajectory smoothness as an indicator rests on the geometry of the test route. The route follows a regular urban street grid, along which the vehicle travels on straight street segments and turns at intersections, so the true trajectory is piecewise straight with near-right-angle turns and contains no high-frequency meandering. Both configurations share the same visual–inertial front end and the same set of receiver measurements and differ only in whether screening is applied, so this difference is mainly attributable to whether the GPS updates are screened before fusion. Two further checks support this interpretation. First, the kinematic quantities inferred under direct fusion are physically implausible for the test vehicle on a narrow urban street, with a maximum speed of 34.82 m/s, about 125 km/h, and a peak acceleration of 333.52 m/s^2^, about 34 g, whereas the proposed screening reduces these values to 7.65 m/s and 59.63 m/s^2^. Second, both configurations traverse the same route and should therefore cover the same distance, yet direct fusion returns a total path length of 1784.16 m against 1697.52 m for the proposed method; the 86.6 m excess is spurious path length produced by high-frequency positional deviations rather than distance actually driven. Trajectory jitter, defined as the standard deviation of the spatial residuals with respect to a moving-average baseline, can therefore be interpreted as a physically grounded indicator of GPS-induced disturbance rather than as a purely aesthetic smoothness measure. We nevertheless acknowledge that the absence of centimeter-level independent ground truth in this scenario remains a limitation.

## 4. Conclusions

This paper presents a GPS/visual–inertial navigation framework to improve localization robustness in dynamic and GPS-degraded environments. Instead of modifying the underlying VIO estimator, the proposed method enhances measurement reliability through dynamic feature masking and GPS measurement screening before estimation.

In the visual front-end, asynchronous YOLOv8 detection and dual-resolution processing identify potentially dynamic regions without blocking the visual–inertial tracking. Visual features within these masked regions are excluded from subsequent estimation. In the dynamic-traffic dataset, dynamic feature masking reduced the horizontal RMSE from 2.074 m to 1.820 m and the maximum horizontal error from 17.823 m to 11.963 m.

For GPS integration, receiver-provided LLA measurements are converted into a local ENU frame and aligned with the VIO coordinate system using an initial temporally corresponding GPS/VIO trajectory segment. Before each GPS update, measurements are accepted only if they satisfy both receiver-reported covariance and NIS-based consistency criteria. In the degraded GPS experiment, the proposed strategy reduced trajectory jitter from 0.0425 m to 0.0218 m and mitigated positional and kinematic disturbances compared to direct GPS/VIO fusion.

The experimental results demonstrate that measurement-level processing at both the visual front end and GPS update stages improves GPS/VIO navigation robustness and reduces trajectory disturbances under the evaluated conditions. The main advantage of the proposed approach is that these mechanisms can be incorporated without modifying the underlying VIO estimator, requiring only receiver-level GPS position and covariance information.

The current validation is limited to ground-vehicle operation in urban traffic and GPS-degraded environments. Additionally, the pseudo-GPS experiment uses a controlled degradation model rather than fully representing real urban GNSS error statistics, and the urban-canyon experiment lacks independent centimeter-level ground truth. Future work will therefore investigate more diverse GPS-degraded scenarios, temporally correlated GNSS errors, and more challenging dynamic environments, including highly maneuverable platforms and rapidly changing object motion.

## Figures and Tables

**Table 1:** Component-by-component comparison with closely related studies.

Method	Dynamic Visual Handling	GNSS Degradation Handling	Fusion Level	GNSS Input	Estimator Modification	Validation
GS-GVINS [3]	None	Raw GNSS tightly integrated optimization	Tight	Pseudorange/Doppler	Dedicated estimator	Urban field data
Sky-GVINS [12]	None	Sky segmentation filters LOS-blocked satellites	Tight	Raw GNSS observations	Dedicated estimator	Urban-canyon field data
DynaVINS [20]	IMU-prior dynamic feature rejection	None (VIO only)	— (VIO only)	—	Modified VIO	Dynamic-scene datasets
Zhai and Yuan [26]	Dynamic-SLAM semantic segmentation	Semantic-based adaptive Kalman filtering (S-AKF)	Loose	Receiver position	Dedicated adaptive KF	KITTI, GNSS-challenged
Adham et al. [27,28]	Smart feature grading + multi-level geometric constraints	Adaptive multilayer VI-GNSS optimization + failure detection	Tight	GNSS observations	Dedicated global VINS	Urban dynamic datasets
Hu et al. [29]	DL features + clustering outlier removal	Fusion-level outlier detection	Tight	GNSS position	Modified fusion estimator	Vehicle field tests
Wang et al. [30]	Semantic mask + stereo epipolar verification	Tightly coupled factor-graph GNSS fusion	Tight	Raw GNSS observations	Dedicated estimator	Stereo field tests
Proposed	Asynchronous YOLOv8 masking, dual resolution	Receiver covariance + NIS two-stage screening	Loose	Receiver-level LLA + covariance	None	Vehicle field tests (dynamic traffic + urban canyon)

**Table 2:** Implementation and parameter settings of the proposed system.

Module	Parameter	Value
Dynamic detection	YOLOv8 variant	YOLOv8n (ONNX, ONNX Runtime, 2 threads)
Dynamic detection	Pretraining source	COCO pre-trained weights
Dynamic detection	Confidence threshold	0.25 (NMS IoU 0.45; detection boxes dilated by 5 px)
Dynamic detection	Dynamic classes	person, bicycle, car, motorcycle, bus, truck (COCO IDs 0/1/2/3/5/7)
Camera	Original resolution	1280 × 1024, monocular
Dynamic detection	Detection input resolution	416 × 416
Async processing	Detector execution rate	13.2 Hz (latest-wins; avg inference latency 20.5 ms)
Async processing	Mask age statistic	No fixed timeout; mean end-to-end mask age 56.2 ms, of which 20.5 ms is mean inference time and the remainder is waiting time at the 13.2 Hz detector rate
GPS/VIO alignment	Motion condition (velocity threshold)	‖v‖ ≥ 0.1 m/s
GPS/VIO alignment	*N*_min_	180
GPS/VIO alignment	*d*_min_	2.0 m
GPS screening	*q*_max_	27.0 m^2^
GPS screening	α	0.05 (χ^2^(3) gate = 7.815)
VIO	Max. tracked features	200 (feature tracking at 21 Hz)
VIO	Feature-tracking thresholds	FAST threshold 20; 5 × 5 grid; min. spacing 25 px; KNN ratio 0.70; MSCKF σpx = 1.5 px

**Table 3:** Positioning accuracy comparison of different VIO and GPS/VIO configurations.

Method	Horizontal RMSE (m)	3D RMSE (m)	Max Horiz Err (m)
NoGPS NoMask VIO	18,867.152	18,933.877	59,294.336
NoGPS Mask VIO	1909.592	1909.737	7943.267
GPS NoMask VIO	2.074	2.133	17.823
GPS Mask VIO	1.820	1.897	11.963

**Table 4:** Kinematic and trajectory smoothness comparison under GPS-degraded conditions.

Metrics	Direct Fusion	Proposed
Path Length (m)	1784.16	1697.52
Max Speed (m/s)	34.82	7.65
Max Acc (m/s^2^)	333.52	59.63
Mean Acc (m/s^2^)	2.26	1.10
Jitter Metric (m)	0.0425	0.0218

## Data Availability

The original contributions presented in this study are included in the article. Further inquiries can be directed to the corresponding author.
